# Abernethy malformation: beware in cases of unexplained hepatic encephalopathy in adults—case report and review of the relevant literature

**DOI:** 10.1259/bjrcr.20170054

**Published:** 2017-11-16

**Authors:** Romeu Duarte Mesquita, Marta Sousa, Filipa Vilaverde, Rosa Cardoso

**Affiliations:** ^1^Department of Radiology, Centro Hospitalar de Entre o Douro e Vouga, Santa Maria da Feira, Portugal; ^2^Department of Radiology, Hospital de Braga, Braga, Portugal

## Abstract

The Abernethy malformation consists of a congenital extrahepatic portosystemic shunt and is believed to be extremely rare in humans. The potential implications of abnormal portovenous shunting and decreased hepatic portal flow are numerous and potentially serious. Although congenital extrahepatic portosystemic shunts are increasingly suspected and diagnosed in specialized centres, much of their clinical presentation and natural history is not fully understood. Symptoms of portosystemic shunt are mainly caused by increased levels of ammonia, which lead to signs of encephalopathy. Therapeutic options depend on the type of shunt and its clinical course, so the classification of the congenital portosystemic shunt is a key finding in these patients.

## Introduction

A portosystemic shunt is defined by the establishment of an atypical connection between the portal vascular system and the systemic circulation. Hence, blood that derives from the abdominal organs, and therefore should follow to the liver by means of the portal vein, is improperly shunted to the systemic circulation. As a consequence, toxins absorbed at the intestinal wall bypass the liver and proceed straight into the systemic circulation. Portosystemic shunts can be congenital or acquired. There are two major categories of congenital shunts, extrahepatic and intrahepatic.

The Abernethy malformation, as the name suggests, was first described by Abernethy and consists of a congenital extrahepatic portosystemic shunt (CEPS).^1,2^ This condition results from persistence of embryonic vessels and is extremely rare in humans. It comprises a group of vascular anomalies of the splanchnic venous system and is characterized by the presence of portomesenteric venous blood draining directly into systemic veins. There are two main types of Abernethy malformations that have been described^3^: Type I (end-to-side shunt) and Type II (side-to-side) shunts. In Type I, there is complete diversion of portal circulation into the systemic blood, apparently with absent intrahepatic portal branches. These Type I shunts are further divided into those in which the splenic vein (SV) and superior mesenteric vein (SMV) drain separately into a systemic vein (Type Ia), and those in which the SV and SMV drain together, after joining to form an abnormally small portal trunk (Type Ib). In Type II shunts, the intrahepatic portal vein is intact but hypoplastic, and some of the portal blood is diverted into a systemic vein (usually the inferior vena cava) through a side-to-side extrahepatic communication. In this type, a variable degree of portal perfusion to the liver remains because there are patent intrahepatic portal veins.

The number of CEPS diagnoses have been increasing in recent years owing to advances in imaging techniques, foetal diagnosis and neonatal mass screening.^4,5^

## Case presentation

A 55-year-old male was admitted to our hospital with clinical evidence of encephalopathy. Physical examination revealed mild jaundice. The patient’s medical history included hypertension, diabetes mellitus, knee arthrosis and resection of a basal cell carcinoma in the nose. The patient was also considered to have hepatic disease, probably related to alcohol consumption. There was a history of alcohol abuse, but the patient referred abstinence from alcohol consumption for the past 6 years.

Liver function testing revealed normal transaminase levels and a total bilirubin level of 2.16 mg dl^–1^ (normal, 0.20–1.20 mg dl^–1^). Serum total protein and albumin were within normal ranges. On haematological studies, no anaemia or coagulopathy was observed. Serum ammonia level was elevated, at 174 μmol l^–1^. Serologic markers for hepatitis B and C were negative; α-fetoprotein was also negative. Laboratory findings on admission are summarized in Table 1.

**Table 1.** Laboratory data on admission.

Haematology	
WBC	4.4 x 10^9^ l^–1^
RBC	5.06 x 10^12^ l^–1^
Hb	16.8 g dl^–1 ^
Ht	46.4%
Plt	161 x 10^9 ^l^–1^
Coagulation	
PT	12.5 sec
INR	1.1
APTT	31.2 sec
Serological examination	
HbsAg	(–)
HbsAb	(–)
HCV	(–)
Blood chemistry	
TP	6.9 g dl^–1^
Alb	3.9 g dl^–1^
T-Bil	2.16 mg dl^–1^
D-Bil	0.8 mg dl^–1^
AST	29 IUl^–1^
ALT	32 IU l^–1^
LDH	296 IU l^–1^
γ-GTP	23 IU l^–1^
ALP	59 IU l^–1^
BUN	28 mg dl
Cr	0.7 mg dl^–1^
CRP	3.5 mg dl^–1^
AFP	2 ng dl^–1^
NH_3_	174.6 μmol l^–1^

γ-GTP, gamma-glutamyl transferase; AFP, alpha-fetoprotein; Alb, albumin; ALP, alkaline phosphatase; ALT, alanine transaminase; APTT, activated partial thromboplastin time; AST, aspartate transaminase; BUN, blood urea nitrogen; Cr, creatinine; CRP, C-reactive protein; D-Bil, direct bilirubin; Hb, haemoglobin; HbsAg, Hepatitis B virus surface antigen; HbsAb, Hepatitis B surface antibody; HCV, Hepatitis C virus; Ht, haematocrit; INR, international normalized ratio; LDH, lactate dehydrogenase; NH3, ammonia; Plt, platelets; PT, prothrombin time; RBC, red blood cell; T-Bil, total bilirubin; TP, total protein; WBC, white blood cell.

**Figure 1.**
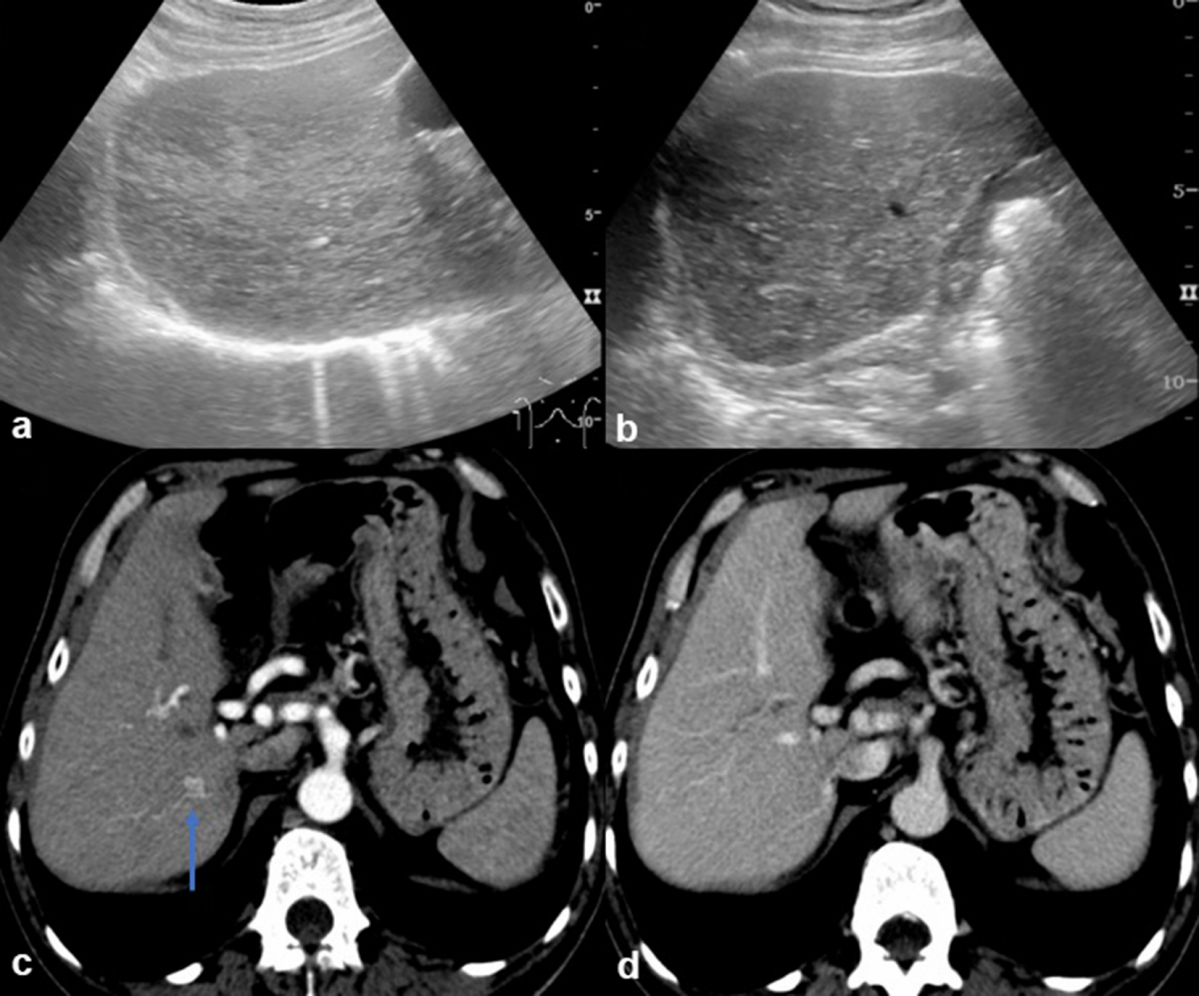
Abdominal ultrasound revealed a normal-sized liver, with a diffusely heterogeneous echostructure suggesting chronic liver disease (a and b). On CT (c and d) the liver is slightly dysmorphic. There is a nodular liver lesion in the right lobe, with CT enhancement pattern in the arterial (c) and portal (d) phases suggesting a benign vascular shunt (arrow in b), which is stable on comparing with multiple previous examinations (not shown).

**Figure 2.**
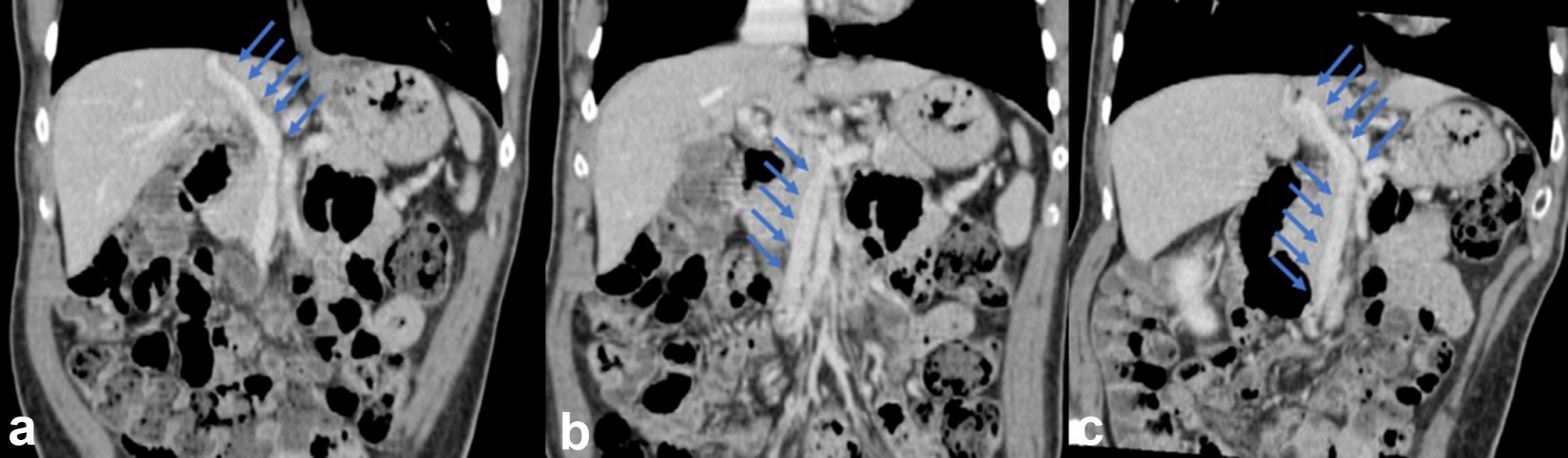
Contrast-enhanced abdominal CT scan. Coronal images showed the presence of an abnormal short portal vein, with a systemic drainage in the inferior cava vein (a), and the superior mesenteric vein and splenic vein draining in the abnormal portal vein (b and c), as depicted by the arrows.

**Figure 3.**
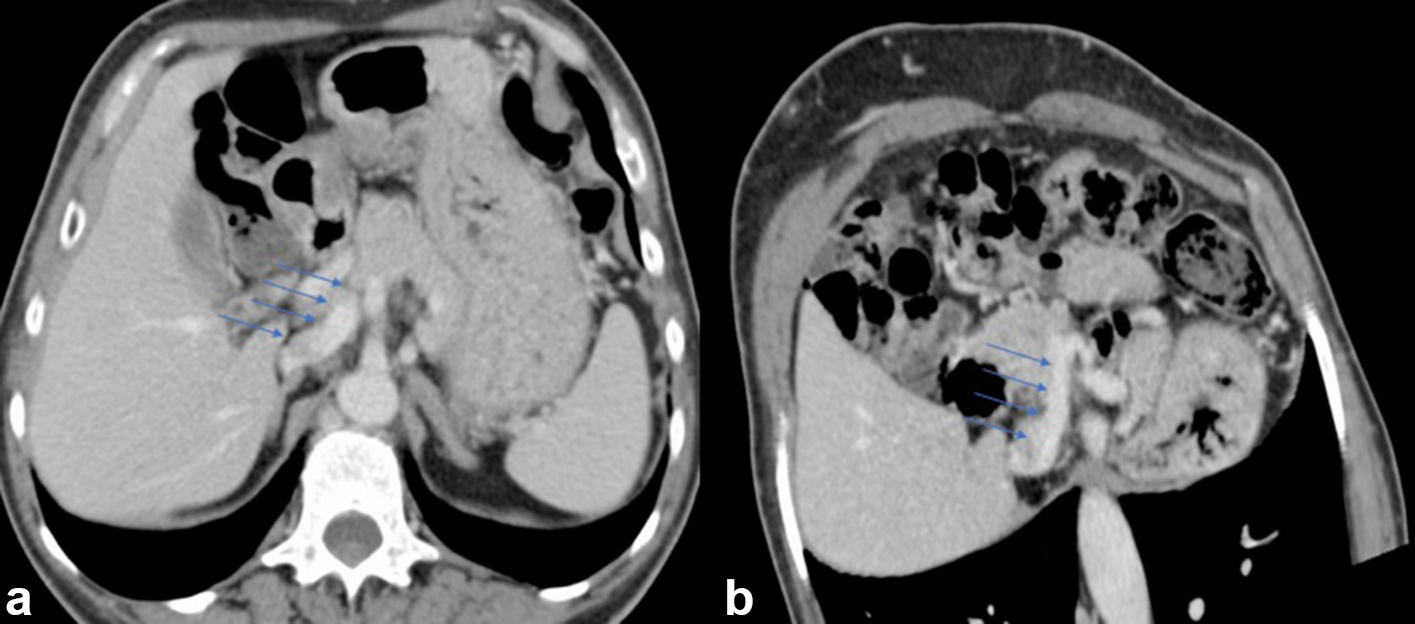
Multiplanar curved reformatted CT images better depicted the short abnormal portal vein draining in the inferior vena cava (a and b).

**Figure 4.**
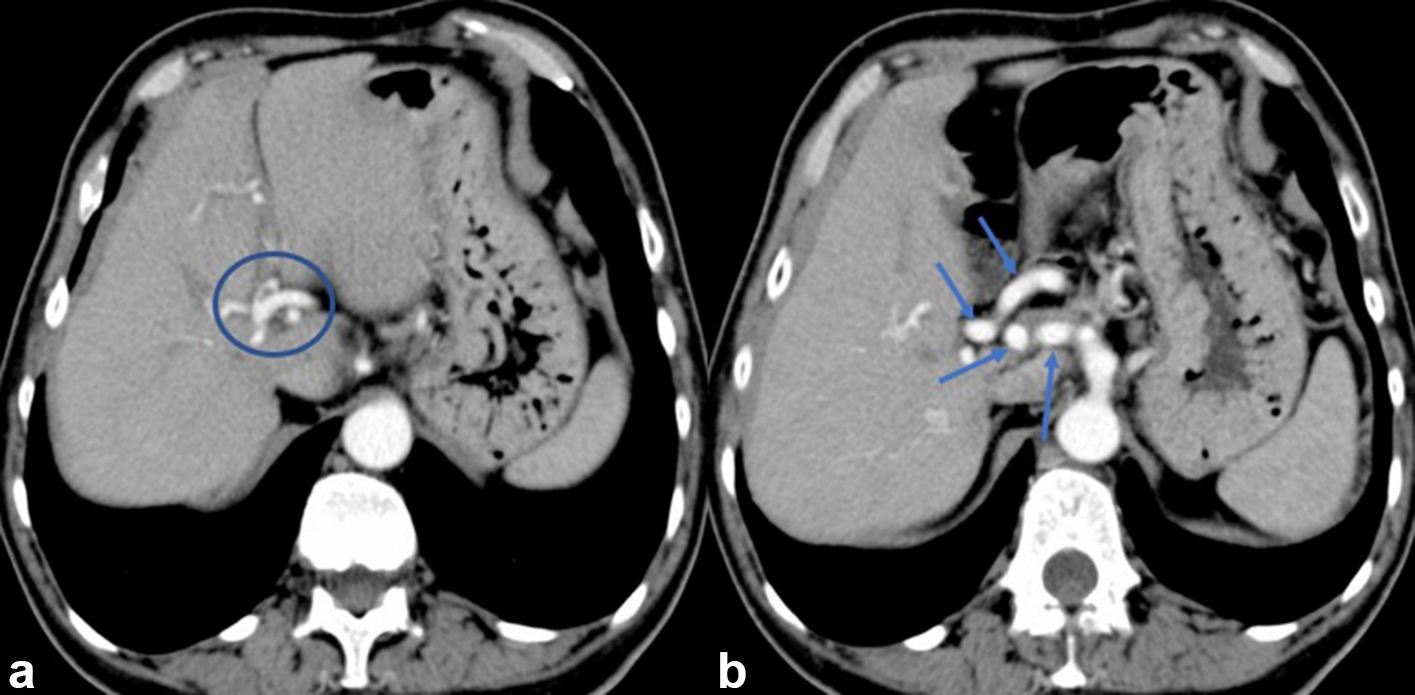
Axial CT images acquired during the arterial phase show a dilated (circle in a) and tortuous hepatic artery (arrows in b).

**Figure 5.**
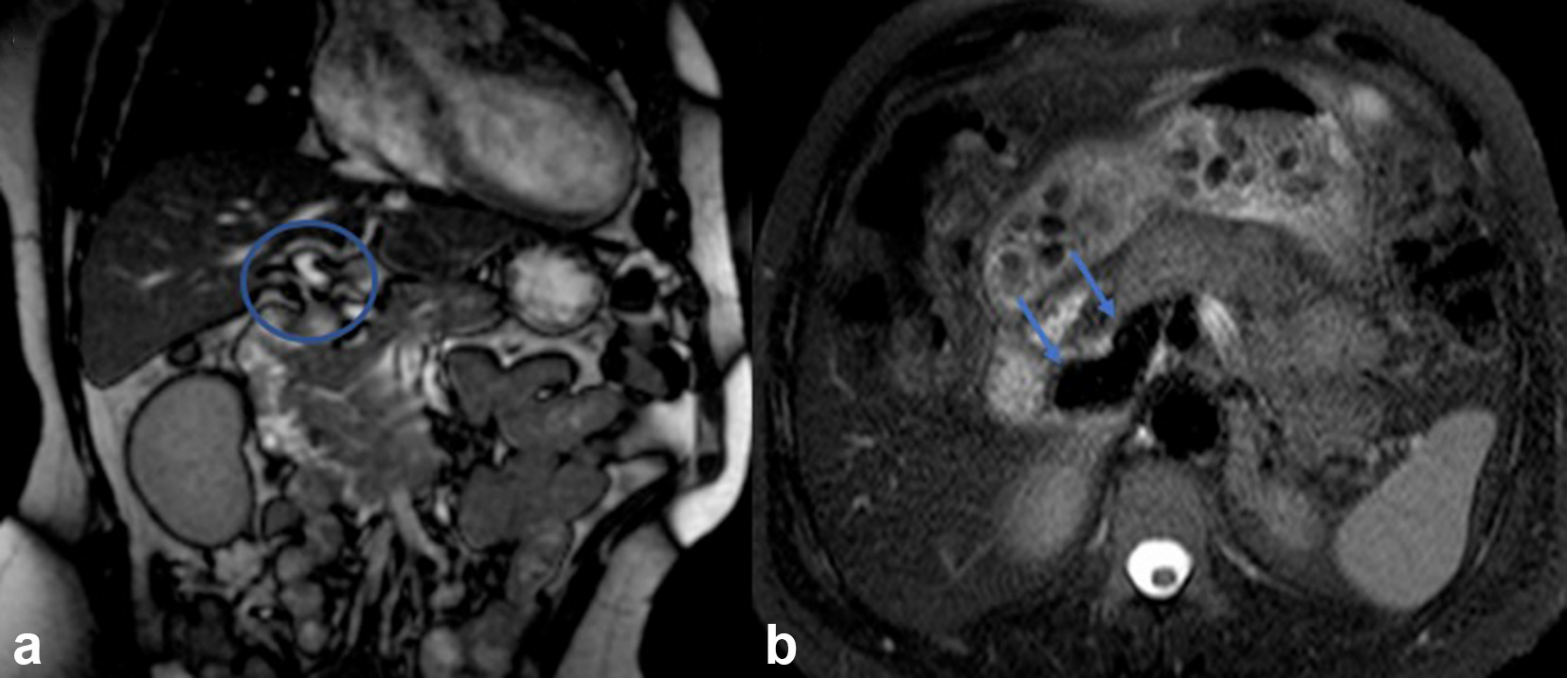
MR images show the presence of the shunt with the inferior vena cava (arrows in b), and abnormal portal vein (circle in a).

Abdominal ultrasonography revealed a diffuse heterogenic echostructure in the liver (Figure 1a, b). Abdominal CT showed the presence of an abnormal short portal vein, with systemic drainage into the inferior cava vein below the liver, and the superior mesenteric vein and splenic vein draining into the abnormal portal vein (Figure 2) (video 1). The venous phase and multiplanar curve reformatted images better depicted this short, dilated shunt vessel (abnormal portal vein) after the confluence of the superior mesenteric vein and splenic vein, and the inferior vena cava (Figure 3). Various nodular hepatic lesions could also be observed, compatible with vascular shunts and regenerative nodules, with the larger nodule presenting a size of 13 mm in diameter. The benign nature of these nodular lesions was confirmed by their stability through at least 7 years. Furthermore, a dilated and tortuous hepatic artery was also detected (Figure 4) (video 2). Although several imaging examinations had been performed during this 7-year time frame, the vascular malformation had not been diagnosed so far. MRI shows the presence of the shunt with similar findings (Figure 5) (video 3).

During a 6-day period of admission in the internal medicine department, the patient received conservative treatment with enema, lactulose, restricted protein intake and branched-chain amino acids. The serum ammonia level improved to 50–60 μmol l^–1^, measured at the day of discharge.

## Diagnosis

According to Ohwada et al^6^ the diagnostic criteria for congenital portosystemic shunt (CPS) include the following:

1. no hypersplenism or portal hypertension
2. no remarkable microscopic changes in liver specimens, such as those of hepatitis, cirrhosis, or idiopathic portal hypertension
3. hypoplastic portal vein with no arterioportal fistula
4. no previous history of abdominal surgery or inflammation.

In the paediatric population, shunts have been detected most often incidentally, usually in the investigation of associated liver dysfunction or cardiac anomalies. The clinical significance of CPS is based specially on these associations, particularly congenital heart defects and nodular liver lesions. However, the clinical diagnosis can be more challenging in older patients, especially in those with coexisting chronic liver disease (much more common) and no early diagnosis of CEPS. Some cases have been misdiagnosed as psychiatric diseases and, subsequently, patients have been hospitalized in psychiatric departments or geriatric institutions.^7^

Shunts can occasionally be detected as the cause of encephalopathy, secondary to chronic hyperammonemia. In CEPS, however, the typically secondary signs of portal venous hypertension such as ascites, varices or splenomegaly are usually absent.^8,9^

For all these reasons, and owing to the fact that the clinical presentations of CEPS can vary extensively, other manifestations cannot be neglected, namely encephalopathy, particularly when diagnosed in older people, as in the case presented in our report.

### Classification

In addition to the most often used anatomical classification, Kobayashi et al^5^ reviewed 136 cases of CEPS and proposed a classification according to the drainage vessel of the portosystemic shunts, which can be viewed as a clinical classification of CEPS. Portal blood flow is then classified by the authors as type A when it is directed into the inferior vena cava (IVC), type B into the renal vein and type C into the iliac vein via the inferior mesenteric vein. According to the authors, this portosystemic shunt classification is more useful to discriminate between the incidences of cardiac anomalies, gastrointestinal bleeding and also prognosis. In this “clinical classification,” the prevalence of cardiac anomalies in patients presenting with type A portal blood flow was slightly higher when compared with patients presenting with other shunt types. However, the presence of lower gastrointestinal bleeding was a considerably more common side-effect in type C patients.

Blanc et al^10^ proposed another classification based on the caval ending of the shunt and CEPS, considering it a useful classification for surgery. In a recent report from 2015 by Kanazawa et al,^11^ a different classification was put forward based on the visualization of the architecture of the intrahepatic portal system. Use of recent techniques in interventional radiology, specifically the shunt occlusion test, allowed them to visualize the intrahepatic portal rami, even in patients previously categorized with absent intrahepatic portal branches (Type I). A total of 18 children with congenital portosystemic shunts who underwent angiography with a shunt occlusion test were classified on the basis of the severity of the hypoplasia of the intrahepatic portal system. According to these authors, it can be useful and can better explain the pathophysiological characteristics of the congenital portosystemic shunt and also play a complementary role in the therapeutic approach and management.

It had been previously stated by Barsky et al that it is highly likely that Type I patients (according to Morgan’s classification), evaluated with other imaging methods, have intrahepatic portal system visualized by angiography under shunt occlusion.^12^ In fact, in their report, Kanazawa et al^11^ demonstrated that almost every case diagnosed with CPS Type I showed visible intrahepatic portal system with the shunt occlusion test. CPS was classified into three types depending on the severity of the hypoplasia of intrahepatic portal system: mild type; moderate type; and severe type. The radiological balloon occlusion test found that a hypoplastic portal system existed in cases with Type I CPS (Morgan’s classification), which had previously been considered to require liver transplantation. A correlation was also established between these differences and the histopathological findings, where the number of portal triads was found to be similar in the mild, moderate, and severe types. The main disparities, however, occurred in the size of the portal triads, which were significantly different in each type. Crescent-shaped portal strictures in a normal-sized portal triad were usually seen in the mild and moderate types. On the other hand, the area of the portal triad was small and the portal vein was not or rarely found in the severe type, suggesting that the intrahepatic portal vascular bed could not accept a proper portal inflow in the severe type, at least immediately after shunt closure.

## Discussion

Type B hepatic encephalopathy describes encephalopathy associated with portal-systemic bypass and no intrinsic hepatocellular disease, according to the consensus group at the 11thWorld Congress of Gastroenterology, where a standardized nomenclature for hepatic encephalopathy was proposed.^13,14^ The major responsible for symptoms of portosystemic encephalopathy is ammonia from the digestive system or elsewhere, which is usually metabolized by the liver in normal conditions, but can lead to signs of encephalopathy when accumulates in the body above a limit threshold value. Owing to portosystemic shunting, whether congenital or not, ammonia enters the systemic circulation and reaches the brain, being toxic to both astrocytes and neurons.^15^ Children rarely present with psychiatric manifestations, but hepatic encephalopathy is rare in infants and children.^9^ Nevertheless, clinical encephalopathy can ultimately result in cognitive disorders and mental retardation, and is considered an absolute indication for treatment in children. Furthermore, a helpful predictor used in screening of CEPS in neonates is elevated levels of galactose,^16^ another toxic metabolite that can bypass the liver.

Different studies report different percentages of patients with encephalopathy associated with congenital shunts, mainly due to the distinct age of the patients studied. In the review by Kobayashi et al,^5^ 18 cases of CEPS (13.2%) were identified as having portosystemic encephalopathy. In their review, despite the fact that prevalence of portosystemic encephalopathy was higher among type A and type B patients and was very rare among type C patients, there were no statistically significant differences between the groups, both in anatomical and clinical classifications. In a series of 97 patients with extrahepatic obstruction of the portal vein, Webb et al^8^ reported clinical and electroencephalographic evidence of portosystemic encephalopathy in 27 (35.5%) cases. However, in this review the patients were initially diagnosed with symptoms of portal hypertension. In fact, Morgan et al^3^ already considered that these malformations would probably go unnoticed in most patients if it were not for its frequent association with the more clinically significant liver and heart anomalies. In initial studies, patients were thought to present with Type II shunts typically at middle or late-middle age. Murray et al,^17^ in turn, contradicted the previously held belief that Type II shunts are typically diagnosed in older children and adults, since they found a younger median age for Type II (2 years versus 10 years for Type I).Studies with larger cohorts are needed to reach a more detailed evaluation.

Despite the different opinions, the initial symptom can often be encephalopathy of unknown cause, with the vascular anomaly detected during investigation, as in the case of our patient. One of the problems for diagnosis and management is that both clinicians and radiologists are generally not sensitized to or aware of this diagnosis and cases with subtle presentations can be extremely challenging. A hallmark of this clinical condition is the elevated ammonia levels. Owing to diminished tolerance of the ageing brain to high ammonia levels, as age progresses it is believed that these patients can become, at some point, symptomatic.^18,19^ Furthermore, the nature of symptoms of portosystemic encephalopathy associated with congenital portosystemic venous shunts depends on the shunt ratio and patient age. Uchino el al^20^ reported that a shunt ratio superior to 60% (determined by portal scintigraphy) was a predisposition for portosystemic encephalopathy in patients where congenital portosystemic venous shunts were present (including patent ductus venosus).^21,22^

Kamiya et al,^23^ in a case report of a patient with congenital absence of the portal vein, proposed that alterations in bacterial intestinal flora, specifically the number of urease-producing microorganisms, could induce a marked decrease in ammonia concentration, urease activity and pH of the faeces, which, in turn, might provide a homeostatic mechanism for maintaining liver function.

Congenital portosystemic venous shunts can be classified by different systems, but the most commonly used is the anatomical classification described by Morgan and Superina,^3^ dividing it into intrahepatic and extrahepatic (CEPS), according to the presence or absence of the intrahepatic portal vein. In CEPS, the anastomoses between the portomesenteric vasculature and a systemic vein are observed before division of the portal vein in the hepatic hilum. The draining systemic vessel can vary, and it can include the renal, iliac, azygos vein or the right atrium, but the most common is the inferior vena cava (portocaval shunt).^17^ While Type I malformation is predominantly described in females, in Type II there is no gender predilection in its prevalence. In a review study of 61 cases, Murray et al found a significant female preponderance (74%) in Type I CEPS, but female preponderance was not significant (54%) in Type II. However, they affirmed that given the likelihood that some cases of Type II CEPS have been misclassified as Type I, the true gender incidence for each type cannot be unquestionably established.^17^

The major challenge both in terms of diagnosis and treatment choice, as exposed in our case report, is in adults that present with long-term chronic hyperammonemia and subclinical encephalopathy. An even more difficult case involves patients that have moderate to high levels of alcohol consumption. In these cases, the clinicians and even radiologists usually do not include a congenital malformation in their differential diagnosis. Furthermore, the anomaly can go completely unrecognized if abdominal imaging examinations are not requested.

Our report exemplifies one of these cases, and our patient presented with a Type Ib shunt (the SMV and SV join to form a short extrahepatic portal vein, which drains into the inferior vena cava).

Recognized associations with Abernethy malformations, apart from hepatic encephalopathy, are hepatic mass lesions, including focal nodular hyperplasia, hepatocellular carcinoma and hepatoblastoma, thought to be related owing to the absence of the portal vein. Other congenital abnormalities particularly associated with Type I shunts are biliary atresia and polysplenia.^24,25^ None of these last referred conditions were diagnosed in our patient.

### Embryogenesis

Development of congenital portosystemic anastomoses can be explained by understanding the origin of their embryological precursors, which have connections that failed to involute between the 4th and 10th weeks of foetal gestation. Embryologically, the anterior and posterior cardinal veins give rise to the systemic veins, which appear as intraembryonic structures. The portal venous system, however, derives from the extraembryonic vitelline and umbilical veins, through an extremely complex process over the course of the first trimester. Aberrations in this process may result in anatomical variations within the portal system. The complicated development of the vena cava, its close relationship with the development of the vitelline veins and the abnormal development of these vessels during this stage may explain the occurrence of the rare congenital extrahepatic portosystemic anastomoses.^24,26^

### Treatment

Therapeutic options depend on the type of shunt and its clinical course, so the classification of the CPS is a key finding in these patients, since it will guide the treatment and determine the correct management. The presence of major complications, such as hepatopulmonary shunt and portopulmonary hypertension, is usually considered an absolute indication for treatment, even when these conditions are mild.^27,28^

Classically, Type I patients are referred for liver transplantation, whereas Type II shunts are amenable to endovascular treatment.^29–31^ The capacity of the hypoplastic portal system to accommodate increased blood flow is a prerequisite for a possible effective endovascular repair. This is usually accomplished by a preprocedure biopsy, which must demonstrate an intact portal intrahepatic system. Multiple studies have shown the liver capacity to reexpand or develop new portal veins following shunt closure.^11,32–34^ The endovascular closure can be done in a single-stage procedure^30^ or using a multistage occlusion technique.^34,35^ In the last case, a liver biopsy previous to the procedure is usually mandatory. Regarding the best choice between single and multistage procedures, Franchi-Abella et al^27^ proposed an algorithm based on a cutoff level of 32 mmHg for the portal vein pressure under shunt occlusion. Franchi-Abella et al^27^ reported that shunt closure should always be performed when complications are present, with the exception of those resolving spontaneously.^36^ They suggested that even when clinical manifestations are not meaningful, the procedure is still recommended when the patient is able to tolerate, because the treatment can be ineffective if performed after important complication occurs. Noteworthy, as previously reported, once patients begin to develop severe shunting, the shunt ratio can quickly increase.^37^

Another aspect of the management is the optimal timing for treatment, for which additional studies are required. Kanazawa et al^11^ also consider that because shunt vessel can expand with advancing age closure of the shunt would be technically harder as the patient gets older. Currently, there are no formal indications for the time of treatment, and therefore, in the majority of the studies published, regarding concerns about paediatric patients, early radical treatments are advised to avoid the development of clinical manifestations related to portosystemic shunt. To our knowledge, patients diagnosed in adulthood, owing to the limited number of cases, have even lesser reported indications.^4,38–40^

In cases of older patients with chronic hyperammonemia and subclinical encephalopathy, the option between closure of the shunt and control with medical therapies remains controversial.^32,33,36,38,41^ Medical management of hepatic encephalopathy with no associated liver disease is usually similar to the treatment of hepatic encephalopathy seen in cirrhotic patients and includes lactulose or non-absorbable oral antibiotics.^7^ However, the necessity for early closure of the shunt and its efficiency in cases where chronic hyperammonemia, and hence subclinical encephalopathy, is controlled with medical therapies need more in-depth studies. This is due to fact that the long-term prognosis of each therapeutic method is currently unclear.^20,42^ Some recent studies report significant recurrence rates of hepatic encephalopathy after embolization of portosystemic shunts.^43,44^ It seems, however, that embolization of portosystemic shunts may be more effective in patients without underlying cirrhosis.^38,39,45^

The patient described in this case report received conservative treatment with enema, lactulose and restricted protein intake. The symptoms progressively improved, and he was discharged after 1 week of admission. The treatment and clinical outcome were established mainly according to improvement in laboratory levels of ammonia. He had had previous similar hospital admissions, managed with a similar approach.

## Learning points

1. Congenital portosystemic shunts are rare vascular anomalies that can cause encephalopathy in the absence of cirrhotic liver disease, only detected in the adulthood.
2. Initial recognition of the presence of non-cirrhotic low-grade/subclinical encephalopathy can be very difficult and the suspicion may arise by the presence of otherwise unexplained signs and symptoms, such as occasional hypoglycaemia.
3. The diagnosis of congenital portosystemic shunts in patients with liver disease is even more challenging, and the occurrence can only be detected by alert clinicians and/or when alterations suggesting portosystemic shunting with no abnormalities of liver function tests are discovered.
4. Abdominal imaging diagnosis plays a central role in these cases and radiologists should be aware of the possibility in a suspicious clinical context.

## Consent

Written informed consent for the case to be published (including images, case history and data) was obtained from the patient(s) for publication of this case report, including accompanying images.
